# Decreased Anti-Tumor Cytotoxic Immunity among Microsatellite-Stable Colon Cancers from African Americans

**DOI:** 10.1371/journal.pone.0156660

**Published:** 2016-06-16

**Authors:** Ranor C. B. Basa, Vince Davies, Xiaoxiao Li, Bhavya Murali, Jinel Shah, Bing Yang, Shi Li, Mohammad W. Khan, Mengxi Tian, Ruth Tejada, Avan Hassan, Allen Washington, Bhramar Mukherjee, John M. Carethers, Kathleen L. McGuire

**Affiliations:** 1 Department of Biology, Molecular Biology Institute, San Diego State University, San Diego, California, United States of America; 2 School of Public Health, University of Michigan, Ann Arbor, Michigan, United States of America; 3 Department of Internal Medicine, Medical School, University of Michigan, Ann Arbor, Michigan, United States of America; Howard University, UNITED STATES

## Abstract

Colorectal cancer is a leading cause of cancer related deaths in the U.S., with African-Americans having higher incidence and mortality rates than Caucasian-Americans. Recent studies have demonstrated that anti-tumor cytotoxic T lymphocytes provide protection to patients with colon cancer while patients deficient in these responses have significantly worse prognosis. To determine if differences in cytotoxic immunity might play a role in racial disparities in colorectal cancer 258 microsatellite-stable colon tumors were examined for infiltrating immune biomarkers via immunohistochemistry. Descriptive summary statistics were calculated using two-sample Wilcoxon rank sum tests, while linear regression models with log-transformed data were used to assess differences in race and Pearson and Spearman correlations were used to correlate different biomarkers. The association between different biomarkers was also assessed using linear regression after adjusting for covariates. No significant differences were observed in CD8^+^ (p = 0.83), CD57^+^ (p = 0.55), and IL-17-expressing (p = 0.63) cell numbers within the tumor samples tested. When infiltration of granzyme B^+^ cells was analyzed, however, a significant difference was observed, with African Americans having lower infiltration of cells expressing this cytotoxic marker than Caucasians (p<0.01). Analysis of infiltrating granzyme B^+^ cells at the invasive borders of the tumor revealed an even greater difference by race (p<0.001). Taken together, the data presented suggest differences in anti-tumor immune cytotoxicity may be a contributing factor in the racial disparities observed in colorectal cancer.

## Introduction

Colorectal cancer (CRC) is one of the most prevalent cancers in the U.S., affecting 1 in 20 Americans during their lifetime. It has the highest incidence among gastrointestinal cancers, affecting over 132,000 Americans in 2015, and it still causes nearly 50,000 deaths per year [1]. Key risk factors include age, family or personal history, environmental factors, inflammation, and ethnicity/race. African Americans (AA) have the highest incidence and death rates for CRC compared to any other race/ethnicity, and have a higher proportion of CRC under age 50 compared with Caucasian Americans (CA) [2, 3]. It is still not clear as to what extent genetic, dietary, lifestyle, socioeconomic, or healthcare issues account for the differences detected in AA.

The idea that the body’s immune system is capable of identifying and destroying cancer has been around some time [4–7]. One challenge the immune system has is the strong immunosuppressive qualities of the tumor microenvironment that limit the potential of immunity in interceding efficiently [8, 9]. Despite this, it is well established in mouse models that the immune system is able to recognize and eliminate primary developing tumors [4–6]. It is also known that cancer patients develop spontaneous adaptive and innate immunity against developing tumors. Studies on CRC have shown both the quantity and quality of the immune response is statistically associated with patient outcome [10–14]. Patients that have a high infiltration of anti-tumor immune cells within and around the tumor have a better prognosis than patients without these cells or those with high infiltration of pro-tumor immune cells, independent of tumor grade and stage [10, 12]. Importantly, the presence of cytotoxic and memory cells within the tumors is predictive of the prognosis of patients with stage I and II disease [15]. Therefore, the type of immune response can influence whether tumor growth is promoted or inhibited and cytotoxic responses dominated by T_Helper_1 (T_H_1) cells and cytotoxic T lymphocytes (CTLs) can be significantly protective—particularly against metastasis—in CRC.

In this study, the possibility that immunity might play a role in the racial disparities observed in CRC is explored using microsatellite-stable (MSS) colon cancer samples. Significantly lower cytotoxic cell infiltration was observed in tumors from AA *vs*. CA patients, strongly suggesting that lower protective immune responses are one biological mechanism contributing to racial disparities in CRC.

## Materials and Methods

### North Carolina Colon Cancer Study (NCCCS)

The NCCCS is a population-based case study with 45% AA, 55% CA patients [16, 17]. The data were collected from 33 counties in North Carolina, and included patients from rural, suburban, and urban population with AA and CA from different socioeconomic backgrounds. Tumor samples were obtained through surgical resection, formalin-fixed, and paraffin-embedded (FFPE), and 5μm slices were affixed to slides. The microsatellite instability (MSI) status was determined for each tumor sample in the NCCCS as described previously [18], using accepted MSI marker loci (BAT25, BAT26, D5S346, D2S123, and D17S250). None of the tumor samples used in this study displayed MSI; i.e. all are of the MSS phenotype. Because the NCCCS tumor bank used in this study is an archived tumor bank, and patient record information was anonymized and de-identified prior to analysis, review by the San Diego State University Institutional Review Board determined these studies were exempt.

**Fig 1:**
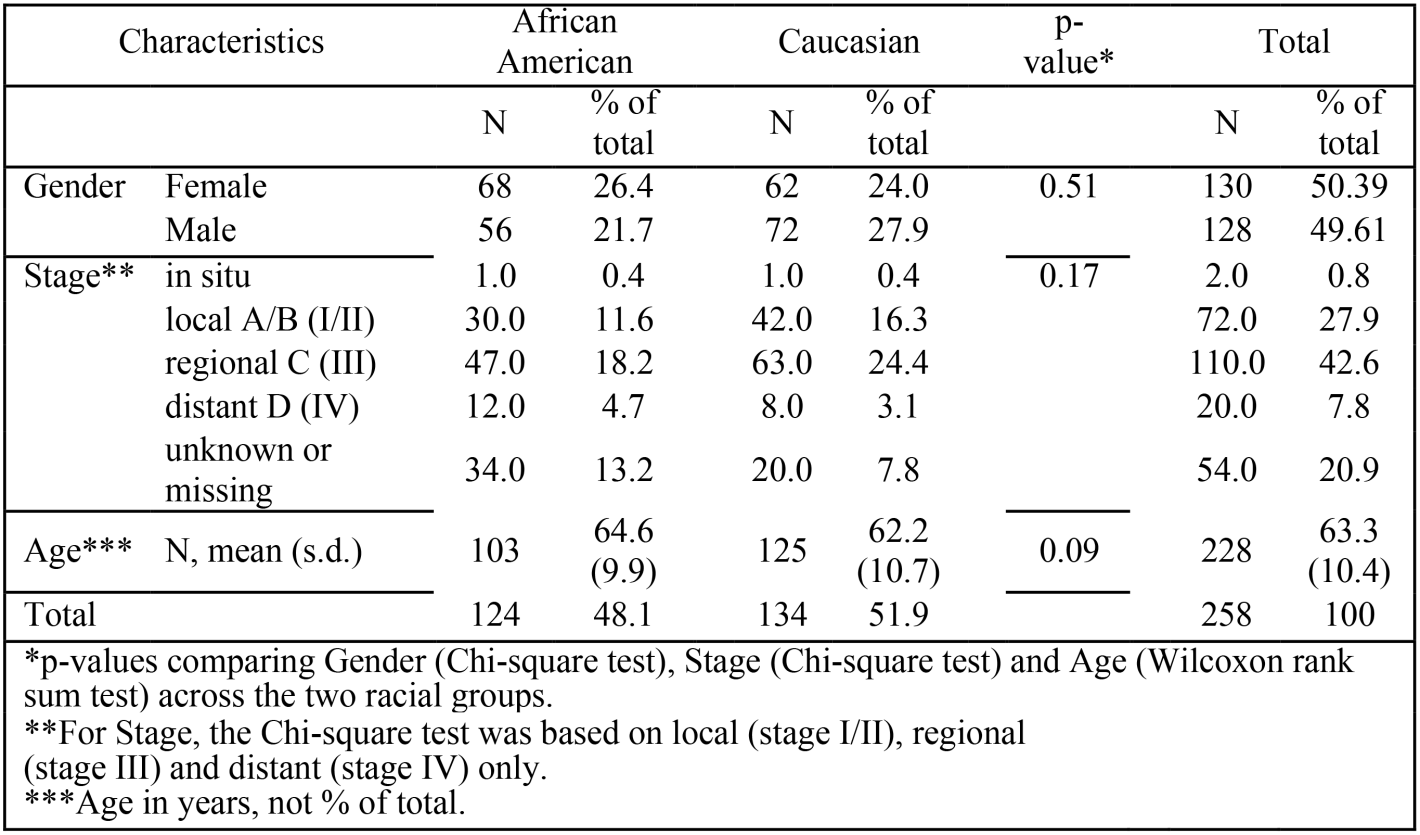
Characteristics of study participants by race. The 258 colon cancer samples from the NCCCS used in this study are described by race, gender, stage and age.

A total of 258 patient colon cancer samples were included in these analyses. No significant differences in the patient demographics, with respect to gender (p = 0.51), stage (p = 0.17) or age (p = 0.09) were found between the AA and CA patients studied (Fig 1).

### Immunohistochemical (IHC) Staining

IHC staining and quantification of CD8^+^ infiltrating cells in these samples has been reported elsewhere [18]. For CD57, GzmB, and IL-17, slides were deparaffinized by soaking in xylene and rehydrated in ethanol followed by water. Antigen retrieval was done in 10mM citrate buffer with 0.05% Tween-20, pH = 6.0 (anti-CD57) or in 1X TE, pH = 8.0 (anti-GzmB) for 10 min in the microwave, or in citrate buffer for 90 sec in a pressure cooker (anti-IL-17). Endogenous peroxidase activity was blocked using 3% H_2_O_2_ (10% for GzmB). Slides were then pre-blocked with 1% BSA in PBS (anti-CD57), 2% human serum in PBS (anti-GzmB), or 5% goat serum/5% BSA (anti-IL-17) for 30 minutes, followed by overnight incubation with specific antibody at 4°C. The antibodies and their dilutions were: mouse anti-human CD57 (1:25 in 1% BSA/PBS; clone TB01; Dako, Carpinteria, CA); mouse anti-human GzmB (1:10 in Dako Antibody Diluent with Background Reducing Components; clone GrB-7; Dako) and rabbit anti-mouse IL-17a (1:50 in 5% Goat serum/5% BSA in PBS; Santa Cruz Biotechnologies #SC-7927). The number of samples stained with each Ab was: 258 for anti-CD8 [18], 245 for anti-CD57, 249 for anti-GzmB, and 234 for anti-IL-17. All tumors were stained for at least two biomarkers using separate sections.

After incubation, the slides were washed in PBS and the LSAB2 kit (Dako #K0673) was used to visualize CD57^+^ and GzmB^+^ cells; HRP-conjugated goat anti-rabbit IgG (Dako #K4002) was used to visualize IL-17^+^ cells. The chromogenic substrate was 3,3’-diaminobenzidine for all stains, and the slides were counterstained with Mayer’s hematoxylin (Sigma-Aldrich #S3022, St. Louis, MO). Lastly, the slides were mounted with Faramount aqueous mounting medium (Dako #S3025) and cover-slipped.

### Microscopy and Data Acquisition

Three high-powered fields (hpfs) with maximum cell infiltration at 100X total magnification were captured for CD57 and IL-17 using a Nikon E600 Teaching microscope mounted with a SPOT QE camera at the Microscopy Core Facility (John and Rebecca Moore’s Cancer Center, UC, San Diego, CA). Six hpfs at 200X magnification were similarly captured for GzmB. Care was taken to avoid overlap of fields. GzmB^+^, CD57^+^, and IL-17^+^ cell infiltration were manually quantified from the hpfs by two independent observers and the counts were averaged if they fell within 10% for GzmB and CD57, or within 15% for IL-17. If not, then a joint reconciliation was performed and the samples were recounted and averaged, as previously described [18–21]. The count used to represent a particular sample for each biomarker was obtained by averaging all of the counts for that sample by both observers. Counts were done for intraepithelial (IE) infiltration (positive cells infiltrating epithelial tumor cell nests) for GzmB and CD57; total intratumoral (IT) cell numbers were determined for all three markers. GzmB-expressing cells were also counted at invasive borders (IB) in the samples that showed an invasive border. IE CD8^+^ cell counts for these samples have been reported elsewhere [18].

### Statistical Analysis

Characteristics of the study populations, stratified by race, were summarized using descriptive statistics in the form of frequencies for categorical variables. For two-sample comparison chi-squared tests were used for categorical variables. Continuous variables were summarized using mean, standard deviation, median, and range. The cell infiltration markers were often heavily skewed and thus were log-transformed. Non-parametric Wilcoxon rank sum tests of unadjusted association on the untransformed raw markers were carried out, whereas adjusted models were constructed using standard linear regression on the log-transformed markers. Interaction of two-way and three-way factors was explored by adding product terms to the linear regression model. Wald tests for significance were used to report the p-values. Mann-Whitney analyses were used to generate p values for the comparison of GzmB^+^ cells at the IB.

## Results

### CD8^+^ and CD57^+^ cells infiltrate human MSS CRC, but do not differ by race

**Fig 2:**
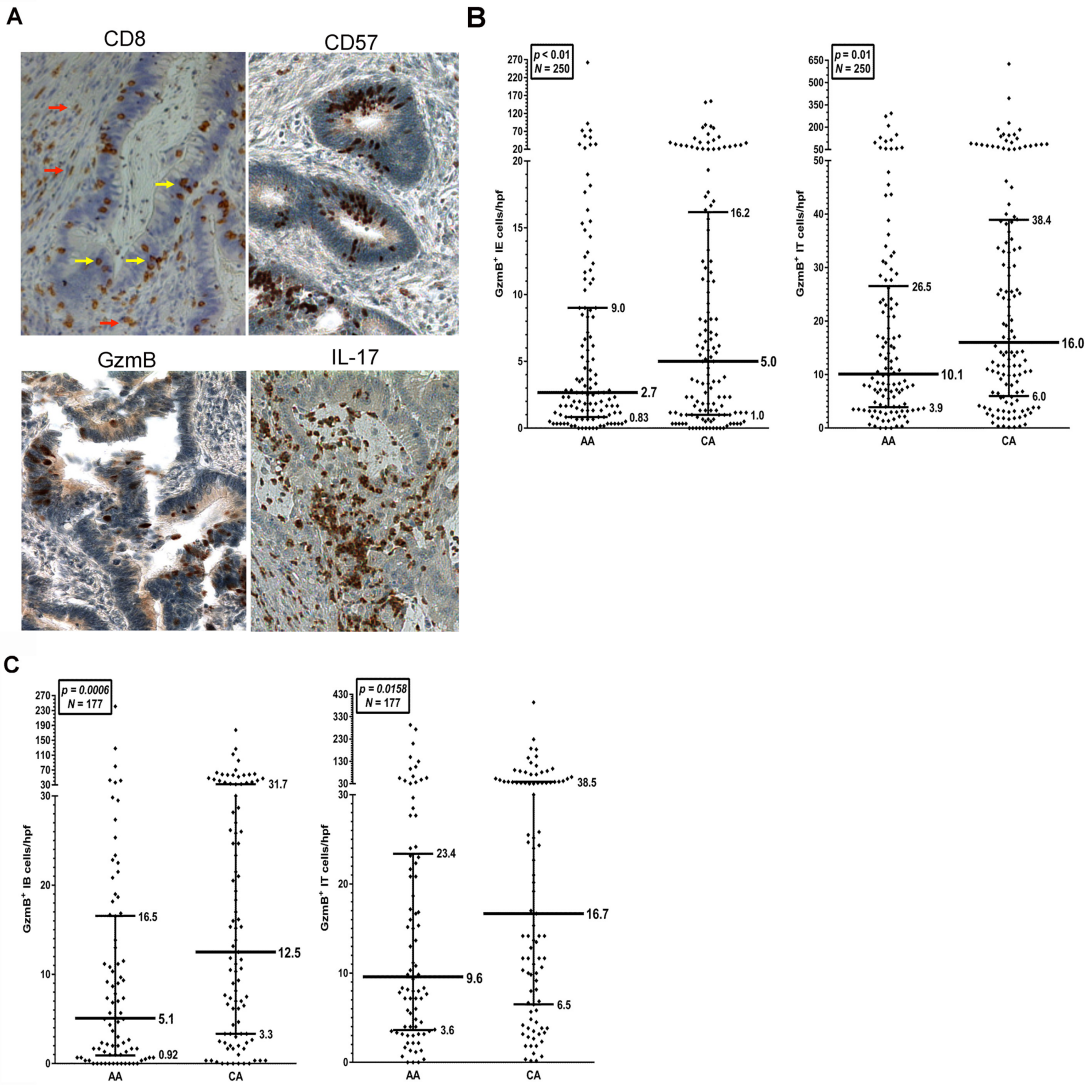
Presence of CD8^+^, CD57^+^, GzmB^+^ and IL-17^+^ cells in CRC. **A**) IHC staining of colon cancer samples from the NCCCS for the markers indicated. Red arrows indicate cells positive for CD8 in the stromal tissue while yellow arrows indicate staining of CD8^+^ cells in the epithelial tumor nests (IE staining). **B**) Distribution of GzmB^+^ cell infiltration in CRC samples. Dot plots of IE and IT GzmB^+^ infiltrating cells in the NCCCS samples stained by IHC. The median cell numbers are indicated with bold horizontal lines, with the interquartile ranges indicated in thinner lines. **C**) Distribution of GzmB^+^ cells at the IB. Dot plots of the IB and IT GzmB^+^ infiltrating cells in the NCCCS tumor samples from Fig 2A and 2B that contained an invasive margin.

**Fig 3:**
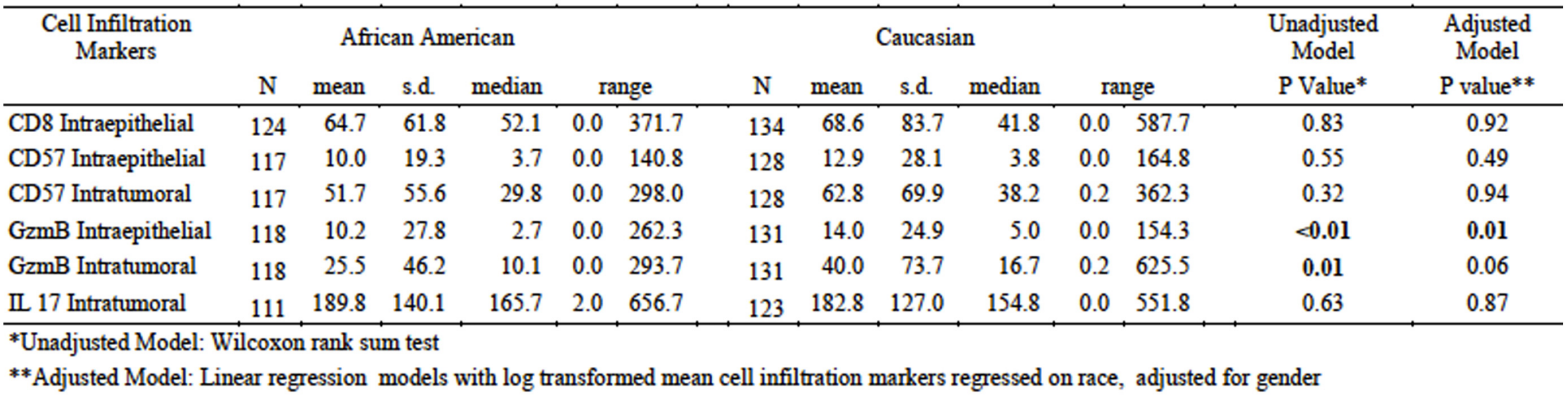
Summary statistics of the six cell infiltration biomarkers by race. The infiltration of cells positive for each of the markers (CD8, CD57, GzmB, and IL-17) are indicated, as is whether the data is for positive cells infiltrating the entire tumor (IT, epithelium and stroma) *versus* the epithelium alone (IE, intraepithelial).

IHC was used to reveal infiltrating immune cells in 258 MSS CRC samples, 124 from AA and 134 from CA patients (Fig 2). The analysis of CD8^+^ cells infiltrating the tumors was limited to the epithelium because previous studies had shown that only IE CD8^+^ cells correlated with better prognosis [19, 20]. As shown in the example in Fig 2A, positively-stained IE cells are easily distinguished from those in the stroma in most adenocarcinoma samples (arrows). However, no significant difference in IE CD8^+^ cell infiltration by race was observed in the total NCCCS samples tested [18], and that remained true for the subset used in these studies (p = 0.83) (Fig 3).

CD57 has been historically thought of as an NK cell marker [21], but more recent analyses have suggested that CD57 is expressed by both mature NK cells [22] and CTLs [23], indicating it is a marker for cytotoxic activity. CD57 was analyzed to determine if tumor infiltrating lymphocytes (TIL) include potentially cytotoxic cells and if their number differs by race. As shown in Fig 2A, CD57^+^ infiltrating cells were observed, but their numbers were much lower than CD8^+^ cells (Fig 3). Both IE and IT infiltration were determined but there were no significant differences observed in either by race (p = 0.55 and 0.32, respectively). This is similar to what was observed with CD8^+^ cells, strongly suggesting that different numbers of potentially cytotoxic infiltrating cells are not seen between AA and CA CRC samples.

### Levels of infiltrating GzmB^+^ cells differ significantly between AA and CA

Cytolytic enzymes like GzmB are expressed by mature CD8^+^ and CD57^+^ cells, and represent a major mechanism for targeted tumor cell lysis by cytotoxic immune cells. GzmB is a serine esterase that activates caspases and apoptosis in target cells, and it is a component of the cytotoxic granules found in mature CTLs and activated NK cells [24]. Previous studies on this and related enzymes have revealed that their presence in tumors correlates with better patient prognosis [12, 19, 25]. Therefore, to verify the cytolytic activity of the TILs seen in these tumor samples, GzmB^+^ cell infiltration was analyzed. As shown in Fig 3, a significant difference in IE infiltration of GzmB^+^ cells was observed by race (p<0.01). AA samples overall had significantly lower GzmB^+^ cells than tumors from CA. There was also a significant difference in IT infiltration of GzmB^+^ cells observed by race in the unadjusted Wilcoxon rank-sum test (p = 0.01), but this significance decreased using an adjusted linear regression model (p = 0.06).

It is interesting that the medians between the two races for GzmB^+^ infiltrating cells were not that different; 2.7 in AA *vs*. 5 in CA (IE) and 10.1 in AA *vs*. 16.7 in CA (IT) (Fig 3). However, in scatter plots, the differences between the two races are more obvious and the upper quartile shows greater differences between AA and CA (9 in AA *vs*. 16.2 in CA (IE) and 26.5 in AA *vs*. 38.4 in CA (IT)). As shown in Fig 2B, there is a paucity of “GzmB high responders” in the AA population studied; i.e. samples with >30 IE GzmB^+^ cells/hpf were significantly reduced in the AA population *vs* what is observed in the CA population. Because none of the samples are MSI tumors, which are known to have higher infiltration of immune cells [18–20, 26], these “high responders” are all patients with MSS tumors, regardless of race.

Previous studies have also shown that cytotoxic cells at the invasive margin correlate with better prognosis in CRC patients [11, 12, 27]. Therefore, the tumor samples stained for GzmB^+^ cell infiltration were reanalyzed at the IB in the samples that contained an IB (n = 177; 82 AA; 95 CA). The IT infiltration still differed by race in this subset of tumor samples (p = 0.016). The difference in GzmB^+^ cell infiltration by race was even more marked at the IB (p = 0.0006), as seen in Fig 2C. The upper quartile shows even greater differences between the two races (5.1 in AA *vs*. 12.5 in CA (IT) and 23.4 in AA *vs*. 38.5 in CA (IB)). Many more “GzmB high responders”, i.e. samples with >15 IB GzmB^+^ cells/hpf, are seen in the CA population. Taken together, these data suggest that decreased anti-tumor cytotoxicity may be one biological mechanism contributing to the racial disparity in patients with MSS CRC.

### Intratumoral IL-17^+^ cell number does not differ by race

One mechanism that has been reported to decrease anti-tumor cytotoxic immune responses is IL-17 [28]. For example, T_H_17 cells were found to be inversely correlated with cytotoxicity in CRC [25]. IT levels of IL-17^+^ cells were analyzed to determine if increases correlated with decreased GzmB expression in AA. IT levels were analyzed in lieu of IE levels, with the rationale that IL-17 is secreted and could affect responses at a distance. There was no significant difference in IL-17^+^ cell numbers (p = 0.63) in AA *vs*. CA (Fig 3). These data show the number of IL-17 producing cells is not likely to be different between the two races. However, because absolute IL-17 levels can’t be determined from these analyses, it is not possible to conclude it plays no role in the observed differences in anti-tumor cytotoxicity.

### GzmB correlates well with the presence of CD8^+^ and CD57^+^ cells

**Fig 4:**
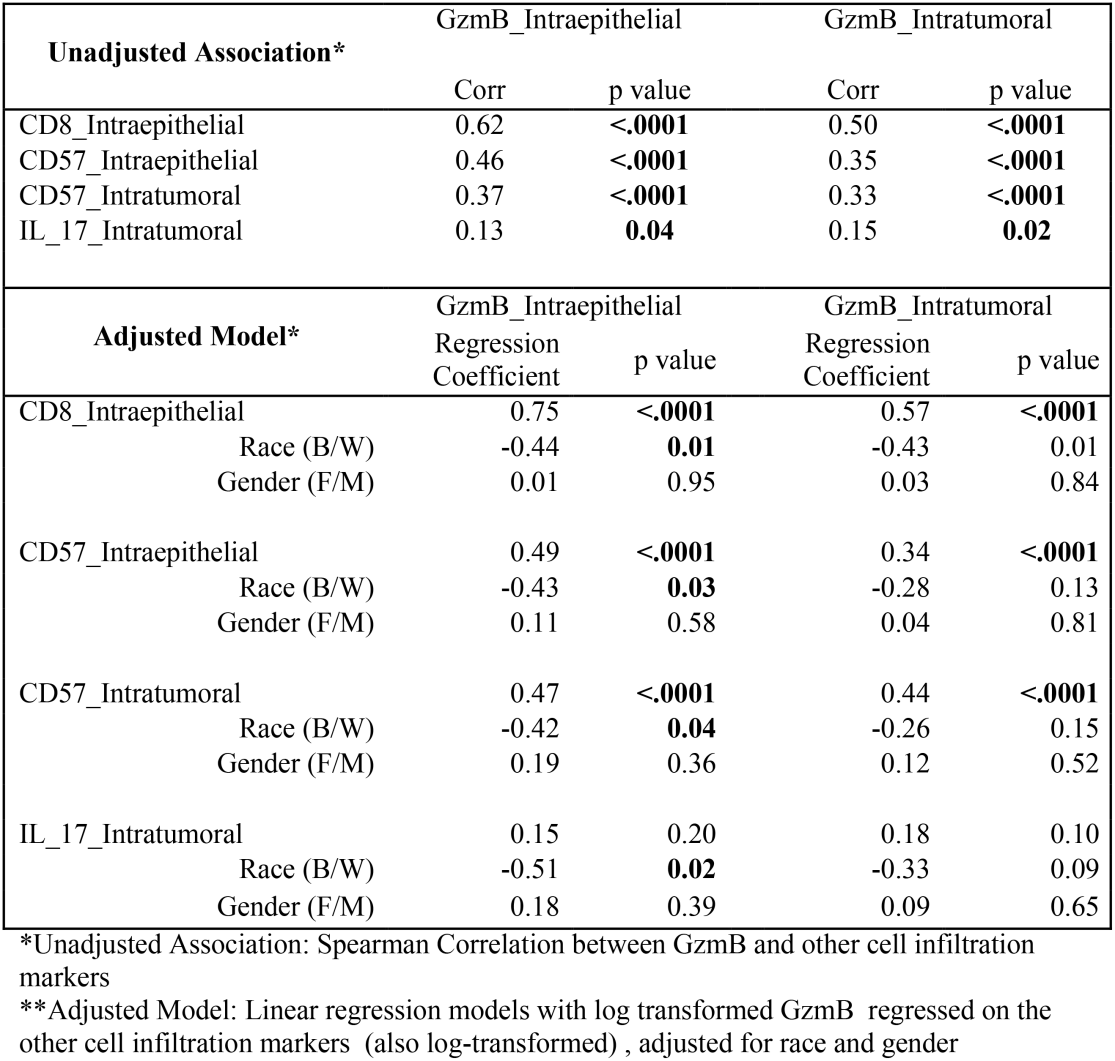
Association between GzmB and other cell infiltration biomarkers. The correlation between GzmB and the other three biomarkers (CD8, CD57, and IL-17) is shown.

To determine if the presence of each immune biomarker positively or inversely correlated with the other immune markers, Spearman correlation tests and linear regression were used on unadjusted and adjusted models, respectively. Not surprisingly, the co-presence of CD8^+^ and CD57^+^ cells strongly correlated in these studies (S1 Table). Strong correlations between GzmB^+^ cells and the presence of both CD8^+^ and CD57^+^ cells (p<0.0001) were also observed (Fig 4). This is logical because these two markers are expressed on the majority of anti-tumor cytotoxic cell types using GzmB as a mediator for cytotoxicity [21,22]. Fig 4 demonstrates there was also a weak positive correlation between the GzmB and IL-17 biomarkers (p = 0.04). A previous study had observed an inverse correlation between IL-17^+^ and cytotoxic cells [25], so this result was somewhat unexpected. Possible explanations are addressed further below.

### No significant correlation between immune biomarkers and tumor stage is observed

**Fig 5:**
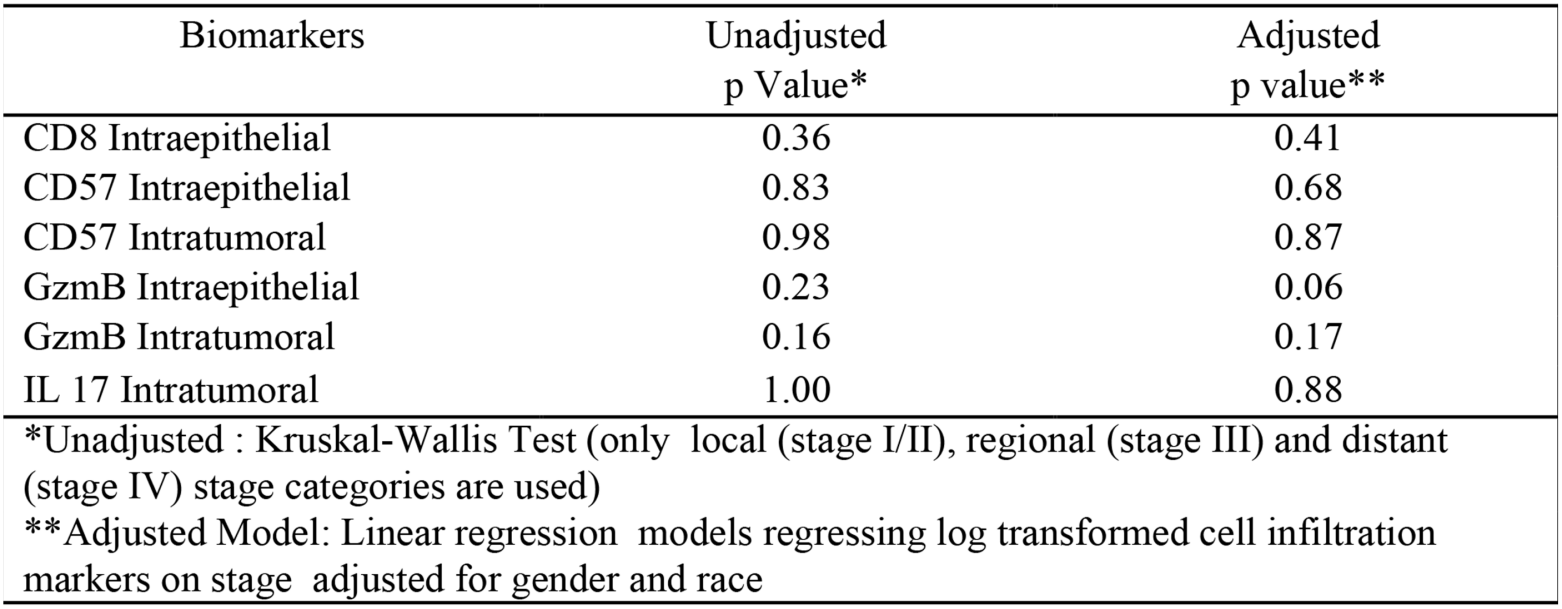
P-values for the unadjusted or adjusted test corresponding to stage differences in the six cell infiltration markers. None of the six biomarkers correlated significantly with tumor staging in the NCCCS samples.

To determine if any of the immune biomarkers correlated with tumor stage, Kruskal-Wallis Tests and linear regression were done on unadjusted and adjusted models, respectively. Fig 5 demonstrates there is no significant association between any of the biomarkers and tumor stage in these samples. It is important to note, however, that the number of advanced stage samples (i.e distant/stage IV) was very low in this study and larger numbers of samples may need to be evaluated to see a significant association. Unfortunately, no follow-up data were collected on the NCCCS and, therefore, no analyses on how these biomarkers relate to disease-free survival in AA *vs*. CA patients were possible.

## Discussion

### Anti-tumor cytotoxic activity, but not CD8^+^/CD57^+^ cell numbers, differ by race

Recent studies on CRC have demonstrated the significant impact anti-tumor immune responses have on the prognosis of patients with this disease [10, 12, 29, 30]. These studies have demonstrated that T_H_1-promoted cytotoxicity significantly correlates with improved prognosis and disease-free survival in CRC patients. It is logical, then, to hypothesize that racial disparities observed in CRC could be, in part, related to differences in anti-tumor immunity. The results shown here demonstrate that the numbers of CD8^+^ and CD57^+^ cells infiltrating MSS CRC do not differ significantly by race, suggesting that the numbers of immune cells that are recruited to the tumors is equivalent by race. However, functional activity does not appear to be equivalent in the two races. Cytotoxic activity, as revealed by GzmB^+^ staining, reveals overall lower levels observed in CRC from AA *vs*. CA. It is interesting that the significant differences between the two races appear to be due to a paucity of AA “GzmB high responders”. This suggests there may be fundamental differences in how AA respond to their tumors, immunologically speaking, such that fewer individuals in this population benefit from strong anti-tumor cytotoxicity. One unfortunate drawback to the NCCCS is a lack of follow-up and the absence of survival data on this population. Hence, the differences in anti-tumor immunity could not be correlated with disease-free survival. Taken together, however, these data support the hypothesis that differences in anti-tumor cytotoxicity are one biological mechanism involved in the racial disparities in CRC.

### CD8^+^/CD57^+^ TIL may not correlate with the expression of cytolytic activity in CRC

Because CD57 is thought to be expressed on both NK cells and CTLs that are highly cytotoxic [22, 23], and CD57^+^ infiltrating cells have been shown to have prognostic significance in CRC [31, 32], one might expect an excellent correlation between CD57^+^ and GzmB^+^ cell numbers in CRC. A strong correlation was observed between the presence of GzmB^+^, CD8^+^ and CD57^+^ cells overall. However, there is a significant difference in GzmB^+^ cell infiltration by race which is not observed for CD57^+^ cells. This suggests that CD57 does not completely correlate as a biomarker for mature cytotoxicity in lymphocytes infiltrating CRC. It is possible CD57^+^ TIL lose their capacity to express cytolytic enzymes in the immunosuppressive environment of the tumor [29]. Another possible explanation is that GzmB is not expressed in cytotoxic CD57^+^ TIL, but this is not consistent with what is known about TIL activity or the expression of this enzyme [23].

### IL-17^+^ cell levels do not differ by race

Many studies have demonstrated conflicting anti- and pro-tumor roles of IL-17 (and T_H_17 cells) in cancer (for review see [28]). The data presented here demonstrate there is no difference in IT IL-17^+^ cell numbers by race, indicating it may play no role in the racial disparity. It is important to note, however, that the number of IL-17^+^ cells analyzed here doesn’t necessarily correlate with the absolute levels of secreted IL-17 present in tumor samples. This could explain why a slight positive correlation between IL-17^+^ and GzmB^+^ cells was observed here when a previous study [25] suggested cytotoxic markers negatively correlate with IL-17 levels in CRC lesions. Future elucidation of total IL-17a protein levels in MSS CRC samples from AA and CA may shed further light on the role of this cytokine in the racial disparities of this disease.

### DNA Mismatch Repair (MMR) defects, MSS tumors, and Immune Checkpoints

We have previously shown that AA CRC patients have half the rate of defective DNA MMR, i.e. MSI, in their tumors when compared to CA [18]. MSI CRCs shed a high number of neoantigens as a result of the faulty DNA repair [33] and they are associated with heavy CD8^+^ cell infiltration regardless of race, but recent evidence shows high levels of immune checkpoint proteins are expressed in these tumors. Immune checkpoint blockage, then, can enhance tumor cell killing in MSI tumors [33]. Together these two studies might suggest that PD-1 blockage may not be as beneficial to AA CRC patients as a whole, since they have fewer of the MSI tumors that respond so well to this immunotherapy. Interestingly, this blockage is not as effective in CRCs that do not display MMR defects (i.e. MSS tumors) even though we have shown here that immune responses occur in at least a proportion of MSS CRCs, particularly in CA patients. That MSS CRCs can have immune infiltration has been confirmed in the literature as well [34]. Perhaps other checkpoint pathways regulate immune responses in MSS tumors. Identifying the immune differences that occur by race may aid the approach to immunotherapy for both types of CRC in the future.

### Summary

The idea that differences in anti- and pro-tumor immune responses might differ by race is not entirely new. For example, Jovov et al. [35] demonstrated in microarray studies that immune processes represent a significant portion of the differential gene expression in CRC from AA and CA. Wallace et al. [36] demonstrated the same in prostate cancer; that immune processes were significantly represented by differential gene expression in CA and AA prostate cancer was confirmed by Kinseth et al. [37]. Here, we have demonstrated that the absolute number of infiltrating CD8^+^ and CD57^+^ cells do not differ by race in MSS CRC, but the level of cytotoxic activity in these cells does, with AA having lower GzmB expression overall than CA. The role of IL-17 in this observation requires further study but the results presented here suggest IL-17^+^ cells do not differ by race. As stated above, these results may have implications for immune checkpoint inhibitors or other therapeutic approaches in the future. Overall, these studies support the conclusion that differences in cytotoxic immunity play an important role in the racial disparities observed in CRC.

## Supporting Information

S1 Appendix Raw counts from both observers for all immune markers (CD8. CD57, IL-17, GzmB) on all NCCCS samples tested. (XLS)

S1 Table Spearman Correlation Matrix across the various cell infiltration biomarkers. (DOC)
