# Supplementary material for: Decreased Anti-Tumor Cytotoxic Immunity among Microsatellite-Stable Colon Cancers from African Americans
Source: PLoS One. 2016 Jun 16;11(6):e0156660. doi: 10.1371/journal.pone.0156660 (PMC4911070; doi:10.1371/journal.pone.0156660)
Supplement: S1 Table — (DOC) [file pone.0156660.s002.doc]

| Supplementary Table S1. Spearman Correlation Matrix across the various cell infiltration biomarkers. | | |  |  |  |  |  |
| --- | --- | --- | --- | --- | --- | --- | --- |
|  |  |  |  |  |  |  |  |
| Spearman Correlation Coefficients | | | | | | | |
|  |  | CD8_Intraepithelial | CD57_Intraepithelial | CD57_Intratumoral | GzmB_Intraepithelial | GzmB_Intratumoral | IL_17_Intratumoral |
| CD8_Intraepithelial | Corr | 1.00 | **0.51** | 0.25 | 0.62 | 0.50 | 0.11 |
|  | Pvalue |  | **<.0001** | <.0001 | <.0001 | <.0001 | 0.09 |
| CD57_Intraepithelial | Corr | **0.51** | 1.00 | 0.63 | 0.46 | 0.35 | 0.07 |
|  | Pvalue | **<.0001** |  | <.0001 | <.0001 | <.0001 | 0.30 |
| CD57_Intratumoral | Corr | 0.25 | 0.63 | 1.00 | 0.37 | 0.33 | 0.08 |
|  | Pvalue | <.0001 | <.0001 |  | <.0001 | <.0001 | 0.21 |
| GzmB_Intraepithelial | Corr | **0.62** | **0.46** | **0.37** | 1.00 | **0.86** | 0.13 |
|  | Pvalue | **<.0001** | **<.0001** | **<.0001** |  | **<.0001** | 0.04 |
| GzmB_Intratumoral | Corr | **0.50** | **0.35** | **0.33** | **0.86** | 1.00 | 0.15 |
|  | Pvalue | **<.0001** | **<.0001** | **<.0001** | **<.0001** |  | 0.02 |
| IL_17_Intratumoral | Corr | 0.11 | 0.07 | 0.08 | 0.13 | 0.15 | 1.00 |
|  | Pvalue | 0.09 | 0.30 | 0.21 | 0.04 | 0.02 |  |
